# High fecundity in an early-diverging ichthyosaur with over ten fetuses

**DOI:** 10.7717/peerj.21620

**Published:** 2026-07-30

**Authors:** Shengxiao Gu, Wei Wang, Xiuti Li, Chun Li

**Affiliations:** 1 Zhejiang Museum of Natural History, Hangzhou, China; 2 Institute of Vertebrate Paleontology and Paleoanthropology, Chinese Academy of Sciences, Beijing, China

**Keywords:** Triassic, Marine reptile, Ichthyosaur, Viviparity, Fetus

## Abstract

The mode of reproduction is a key aspect for an organism to adapt to its environment. However, understanding the reproductive strategies in extinct animals from deep time remains challenging, due to the scarcity of fossils providing such information. Although viviparity is well-documented across various ichthyosaur lineages, our understanding of the reproductive characteristics of early-diverging Triassic taxa is still limited, particularly regarding their litter sizes. Here, we describe an exceptionally preserved gravid specimen of the Middle Triassic ichthyosaur *Mixosaurus* from southwestern China, containing at least eleven fetuses. This represents the largest litter sizes ever recorded in a Triassic ichthyosaur, and even one of the highest numbers of fetuses known in all ichthyosaurs. The well-articulated fetal skeletons exhibit consistent developmental stages and advanced ossification, indicating a late prenatal or perinatal state. Their variable body orientations further suggest the absence of a fixed head-first or tail-first birth posture. The presence of over ten fetuses in *Mixosaurus* demonstrates that high fecundity, potentially indicative of a more r- than K-selection reproductive strategy, had evolved early in ichthyosaur evolutionary history. This reproductive trait may have been a significant factor contributing to the success of this ichthyosaur group with enormous populations and widespread distribution across Northern Hemisphere during the Triassic.

## Introduction

Ichthyosaurs represent an iconic lineage of Mesozoic marine reptiles that became fully adapted to the marine ecosystems. Viviparity has been widely known among multiple ichthyosaur taxa from the Triassic through the Cretaceous (*e.g.*, Maxwell & Caldwell, 2003; Motani et al., 2014). Giving birth to live young is considered to have conferred a significant adaptive advantage by enabling a completely aquatic life history, thereby eliminating the necessity for terrestrial reproduction (Miedema et al., 2023a).

The first scientifically documented case of viviparity in ichthyosaurs was reported in a specimen from the Early Jurassic in the United Kingdom, which preserved a fetus within the pelvic region of an adult individual (Pearce, 1846) identified to be *Ichthyosaurus somesertensis* (Massare & Lomax, 2018), following the initial discovery of gravid female of *Stenopterygius* (SMNS 2) in 1749 (Miedema et al., 2023a). The initial debates centered on whether the small associated skeletons represent fetuses or resulted from predation or cannibalism. Clarifying evidence emerged from the abundant and exquisitely preserved fossil remains of *Stenopterygius* from the Early Jurassic of Holzmaden and surrounding areas in southern Germany (McGowan, 1979). The juvenile skeletons were conclusively identified as fetuses based on a suite of taphonomic and morphological criteria: their consistent preservation within the maternal uterine area rather than the gastric cavity; their typically parallel or curled posture, contrasting with the disarticulated state expected of ingested preys; and the often uniform head-to-tail and tail-to-tail alignment between the juvenile and the mother is inconsistent with a predation scenario (Gingerich et al., 2009), where head-first swallowing is the norm for the prey instead of the fetuses (Böttcher, 1990). Furthermore, the excellent articulation and lack of digestive erosion on these small skeletons argue strongly against their origin as gastric contents, which typically show signs of disarticulation and acid etching in ichthyosaur fossils (Serafini et al., 2025).

So far, there is uncontroversial fossil evidence for viviparity in at least eleven genera of ichthyosaurs spanning from the Early Triassic to the Early Cretaceous: *Chaohusaurus* (Motani et al., 2014) from the Early Triassic; *Mixosaurus* (Brinkmann, 1996; Miedema et al., 2023a), *Besanosaurus* (Dal Sasso & Pinna, 1996), and *Cymbospondylus* (Klein et al., 2020) from the Middle Triassic; *Shonisaurus* (Camp, 1980; Kelley et al., 2022) and *Qianichthyosaurus* (Wang et al., 2008) from the Late Triassic; *Ichthyosaurus* (Deeming et al., 1993; Lomax & Sachs, 2017; Massare & Lomax, 2018), *Leptonectes* (Lomax & Massare, 2012), and *Stenopterygius* (McGowan, 1979; Böttcher, 1990) from the Jurassic; *Maiaspondylus* (Maxwell & Caldwell, 2003), *Platypterygius* (Kear & Zammit, 2014; Stinnesbeck et al., 2014), and *Myobradypterygius* (Pardo-Pérez et al., 2024) from the Cretaceous. In these previous studies, the viviparous reproduction of ichthyosaurs has been discussed, along with the cranial ontogeny of ichthyosaur fetuses (Miedema & Maxwell, 2022; Miedema et al., 2023b) and the fetal orientations of tail-first or head-first in parturition (McGowan, 1979; Gingerich et al., 2009; Miedema et al., 2023a; Motani et al., 2014). The fetal number has been discussed in detail in *Stenopterygius* (McGowan, 1979; Böttcher, 1990) but less investigated in other above ichthyosaurs probably due to the scarcity and poor preservation of the fetal remains. Based on the previous data, the largest-reported litter size of ichthyosaurs is eleven in *Stenopterygius* (Böttcher, 1990), and there are up to eight fetuses in an unidentified Jurassic ichthyosaur (Boyd & Lomax, 2018), while other genera only had a few, normally no more than three, fetuses at the same time. However, whether other ichthyosaurs, especially the early diverging groups in the Triassic, could produce large litters remains unclear.

Here, we report a complete specimen of an adult female assigned to *Mixosaurus panxianensis* from the early Middle Triassic of southwestern China, which preserved at least eleven fetuses respectively articulated and largely *in situ*. Our findings supplement further morphological information on the anatomy of this *Mixosaurus* species, and more significantly, provide fresh insights into the reproductive biology of early-diverging ichthyosaurs and the developmental stages of multiple fetuses within a single gravid individual.

## Materials & Methods

**Figure 1:**
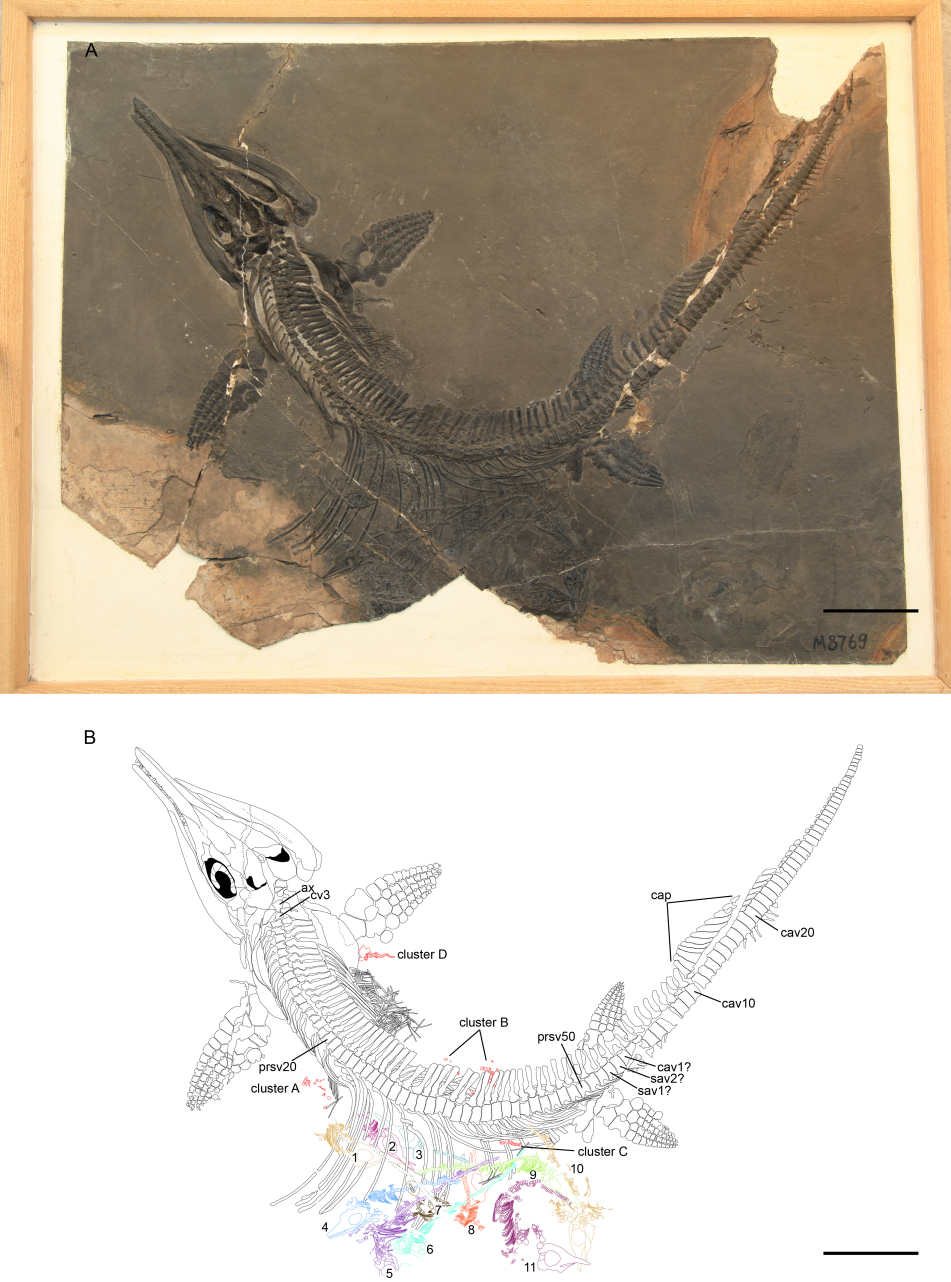
Overview of *Mixosaurus panxianensis* ZMNH M8769. Photograph (A) and outline drawing (B). Abbreviations: ax, axis; cap, caudal peak; cav, caudal vertebra; cv, cervical vertebra; prsv, presacral vertebra; sav, sacral vertebra. Scale bar is 10 cm.

Specimen ZMNH M8769 (Fig. 1), permanently deposited in Zhejiang Museum of Natural History (ZMNH) in Hangzhou, China, comprises a dark-grey limestone block containing a well-preserved adult ichthyosaur alongside eleven ichthyosaurian fetuses and some clusters of vertebral elements. This specimen was collected from Panzhou County, Guizhou Province in southwestern China from the Guanling Formation of the early Middle Triassic (Anisian). It was prepared by professional technicians in the Institute of Vertebrate Paleontology and Paleoanthropology (IVPP) of Chinese Academy of Sciences. The specimen underwent meticulous preparation under the supervision of the authors for scientific research and exhibition, and was reinforced with plaster and equipped with wooden frames. It was prepared with a top-down approach in the plane exposed in the quarry when it was collected.

## Results

### Systematic paleontology

**Table:** 

Ichthyosauria de Blainville, 1835
Mixosauridae Baur, 1887
*Mixosaurus* Baur, 1887
*Mixosaurus panxianensis* Jiang et al., 2006

**Diagnosis** Jugal with short posteroventral process; external contact between jugal and quadratojugal absent (Jiang et al., 2006); anterior teeth relatively large; ratio between height and length in middle caudal centrum over 2.0 (Fang, Wolniewicz & Liu, 2024).

**Holotype** GMPKU-P-1033 (Jiang et al., 2006)

**Referred specimen in this study** ZMNH M8769, a nearly complete skeleton (only the tail tip lost) preserved in dorsal view with the total body length of 112.2 cm and the skull length of 21.6 cm (Fig. 1). This specimen can be assigned to *Mixosaurus* because it possesses the following autapomorphies: premaxilla posteriorly pointed, lacking supranarial process; dorsal margin of orbit formed by supraorbital crest; skull roof distinguished by long sagittal crest on nasal, frontal, and parietal; enlarged anterior terraces of upper temporal opening reaching nasal; mid-caudal vertebral centra with significant height increase; the circumorbital bones consist of lacrimal, prefrontal, postfrontal, postorbital and jugal. It can be further assigned to *M*. *panxianensis* based on the diagnosis above, as well as the same locality and the adjacent stratum layer comparable to the holotype.

**Figure 2:**
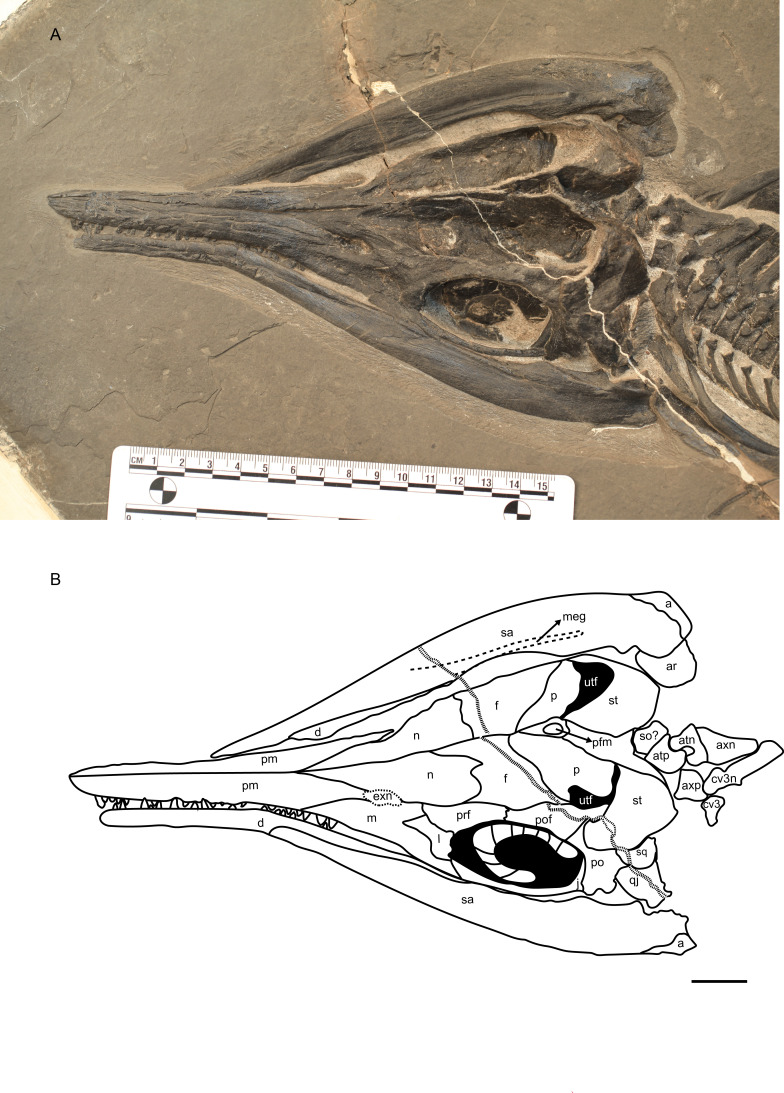
Skull of *Mixosaurus panxianensis* ZMNH M8769. Photograph (A) and outline drawing (B), dashed lines crossing the right lower jaw and the skull table represent cracks. Abbreviations: a, angular; ar, articular; atp, atlas pleurocentrum; axp, axial pleurocentrum; atn, atlas neural arch; axn, axial neural arch; cv3, cervical vertebra 3; cv3n, cervical vertebra 3 neural arch; d, dentary; exn, external naris; f, frontal; j, jugal; l, lacrimal; m, maxilla; meg, Meckelian cartilage grove; n, nasal; p, parietal; pfm, parietal foramen; pm, premaxilla; po, postorbital; pof, postfrontal; prf, prefrontal; qj, quadratojugal; sa, surangular; so, supraoccipital; sq, squamosal; st, supratemporal; utf, upper temporal fenestra. Scale bar is two cm.

### Description

Skull, mandible, and dentition:

The skull, with a total length of 22.0 cm, is preserved in dorsolateral view (Fig. 2). Although several cranial elements are unexposed, the skull is largely complete. Additionally, some remaining bones are fragmented posteriorly, dorsoventrally compressed, and consequently difficult to identify definitively. The left orbit can be discernible, which is slightly deformed with an incomplete sclerotic ring preserved. The general state of preservation is comparable to that of the holotype (GMPKU-P-1033) of this species, which also exhibits a crushed skull roof (Jiang et al., 2006). The ventral cheek embayment is formed by the jugal, postorbital, squamosal, and quadratojugal. The jugal possesses a small posteroventral process. The postorbital is intercalated between the jugal and the quadratojugal with no contact between the two latter elements. The dentition is partially preserved and exposed, with at least 30 teeth observable on the premaxilla and dentary. The majority of these teeth display distinct longitudinal enamel striations. There are 12 premaxillary, 4 maxillary, and 14 dentary teeth exposed, while some of the most posterior teeth on the maxilla and dentary are obscured or lost. The anterior teeth are pointed, slender, and conical, whereas the posterior teeth are blunt-conical with slightly rounded crowns but not molariform. All of the teeth develop longitudinal striations on their crown surfaces. This dental morphology is more consistent with the previous description of other *Mixosaurus* specimens (Brinkmann, 1998; Schmitz et al., 2004; Jiang et al., 2006; Fang, Wolniewicz & Liu, 2024) compared to that in *Phalarodon* (Jiang et al., 2007; Roberts, Sjøholt Engelschiøn & Hurum, 2022), when there is possible intraspecific variation in mixosaurid species.

Vertebral column:

Including the atlas and the axis, the specimen preserves 108 fully articulated vertebrae representing the nearly complete series of the vertebral column, except the most terminal caudal vertebrae. There are about 52 presacral vertebrae, likely 2 sacral vertebrae according to their slightly larger centra and shorter ribs compared to the dorsal elements, and at least 54 caudal vertebrae starting from the anterior ones developing chevrons (Fig. 1). The centrum height is notably increased in the mid-caudal region compared to the presacral ones, which is a diagnostic character in the genera *Mixosaurus*. The axial neural spine is anteroposteriorly broader but dorsoventrally shorter than that of the third cervical vertebra. Most neural arches carry tall neural spines except the distalmost caudal vertebrae. From the anterior to the posterior presacral region, the neural spines exhibit a slight posterior inclination. In the mid-caudal vertebral series, there is a clear caudal peak formed by the anticlination of neural spines (Fig. 1). Ribs are present in all of the precaudal vertebrae, and a few anterior caudal vertebrae develop both ribs and chevrons, while most of the caudal vertebrae possess only chevrons with no rib. The ribs from about the 22nd to the 45th presacral vertebrae are exposed in different direction compared with other ribs, and generally outline the region where the fetal skeletons are preserved (Fig. 1).

**Figure 3:**
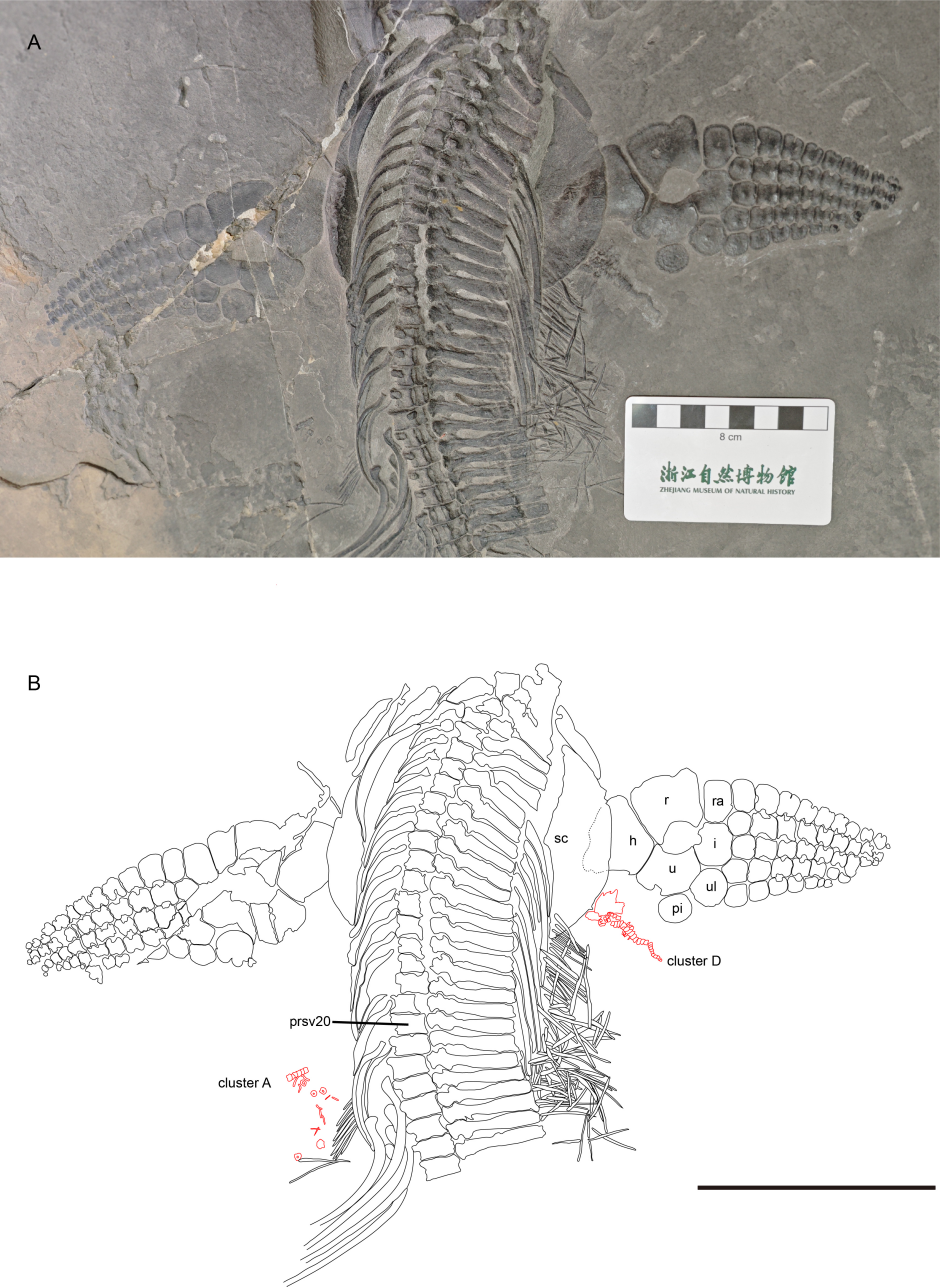
Pectoral girdle, forelimb, and fetal clusters of *Mixosaurus panxianensis* ZMNH M8769. Photograph (A) and outline drawing (B). Abbreviations: h, humerus; i, intermedium; pi, pisiform; prsv, presacral vertebra; r, radius; ra, radiale; sc, scapula; u, ulna; ul, ulnare. Scale bar is 10 cm.

Pectoral girdle and forelimb:

Most parts of the pectoral girdle are obscured by the overlying vertebral column and matrix (Fig. 3). The scapula exhibits a broad, round, and plate-like dorsal region that overlaps the proximal end of the humerus. Both forelimbs are preserved in dorsal view, while a crack runs across the skull, mandible, and left forelimb. The right forelimb is almost complete and easily observable. The humerus is subequal in length and width, and it develops a broad distal end with the articular surface to the radius distinctly larger than that to the ulna. The anterior margin of the radius bears only one deep notch, which is different from the two notches observed in the holotype of *M*. *panxianensis* (Jiang et al., 2006) but intraspecifically variable in this species (Zhou et al., 2022). The forelimb exhibits four carpals, including the radiale, intermedium, ulnare, and pisiform. The intermedium has a proximal concavity. The morphology of carpals, metacarpals, and phalanges with hyperphalangy is comparable to that described for specimens of *M. cornalianus* (Motani, 1999).

**Figure 4:**
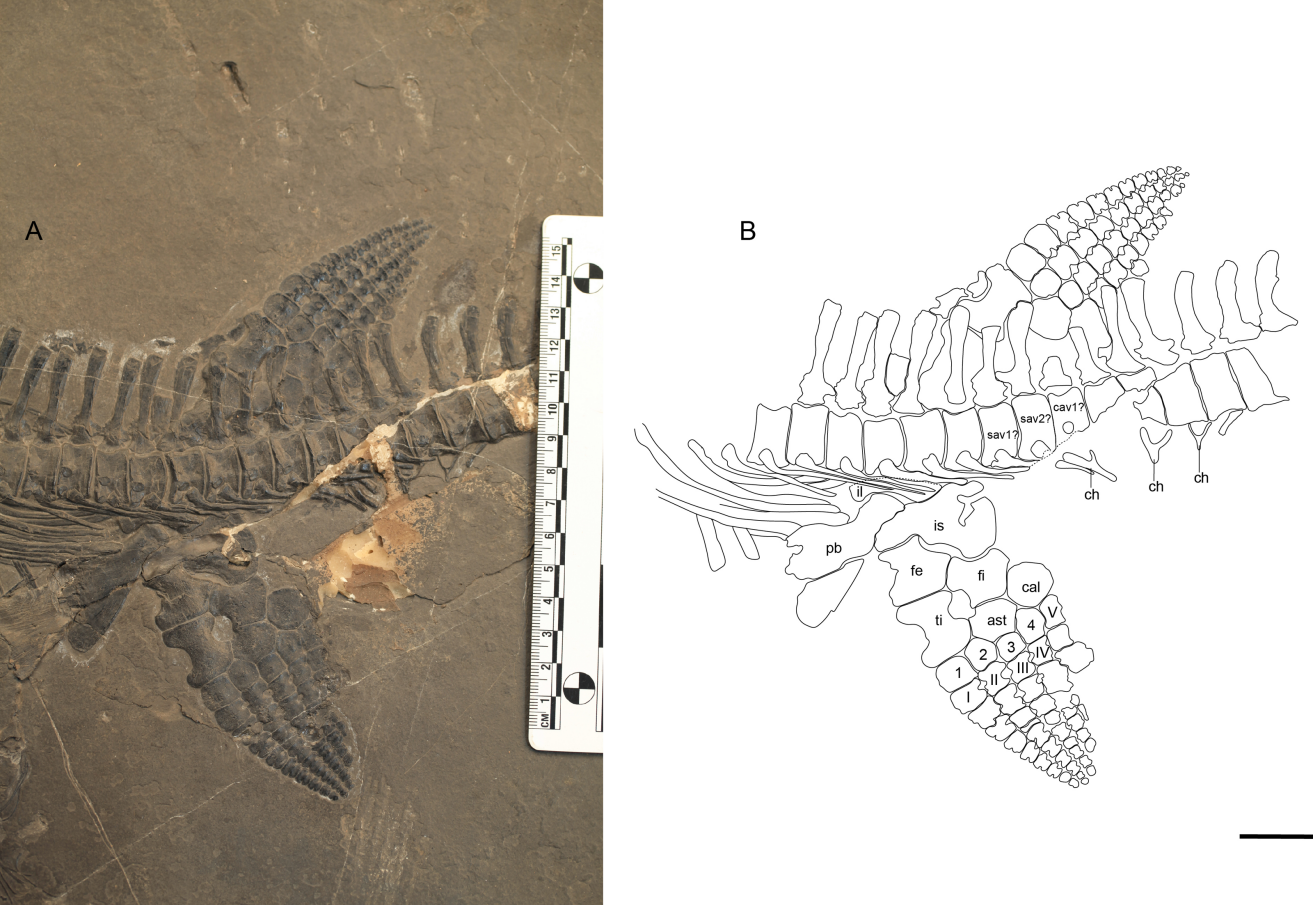
Pelvic girdle and hind limb of *Mixosaurus panxianensis* ZMNH M8769. Photograph (A) and outline drawing (B). Abbreviations: ast, astragalus; cal, calcaneum; ch, chevron; fe, femur; fi, fibula; il, ilium; is, ischium; pb, pubis; sav, sacral vertebra; ti, tibia; 1–4, distal tarsals; I–V, metatarsals. Scale bar is two cm.

Pelvic girdle and hind limb:

The left half of the pelvic girdle is articulated and exposed (Fig. 4). Although partially damaged, the pubis and the ischium can be identified (Fig. 4). The ilium is partially covered by the overlying ribs and centra, and it is a bar-like element similar to that in the paratype of *M*. *panxianensis* (Jiang et al., 2006). Both the left and right hind limbs are nearly completely preserved in dorsal view. The straight articular facet of the femur for the tibia (16.0 mm) is about twice the width of the concave fibular facet (8.3 cm), which corresponds to the neotype of *M. cornalianus* and the paratype of *M*. *panxianensis* (Jiang et al., 2006). The tibia and the fibula enclose a broad spatium interosseum and are distally separated by the astragalus. The tibia possesses a well-defined shaft with subequal proximal and distal widths, and is slightly narrower than long. The fibula is distally much broader than proximally. The fibula articulates with both the astragalus and the rounded calcaneum. The astragalus possesses a proximal notch. Distal to these elements are four distal tarsals, with tarsal 1 being slightly larger than the other three. The shape of the distal tarsals is roughly squared (tarsals 1 and 3) or pentagonal (tarsals 2 and 4). All five metatarsals are all preserved. The leading edge of metatarsal I is convex, while metatarsals II, III, and IV bear distinct anterior and posterior notches. One side of metatarsal V is damaged and could not be further described. The right pes is better preserved, in which up to ten phalanges are present in the digit I, nine in the digit II, eight in the digit III. All of these phalanges show both anterior and posterior notches.

**Figure 5:**
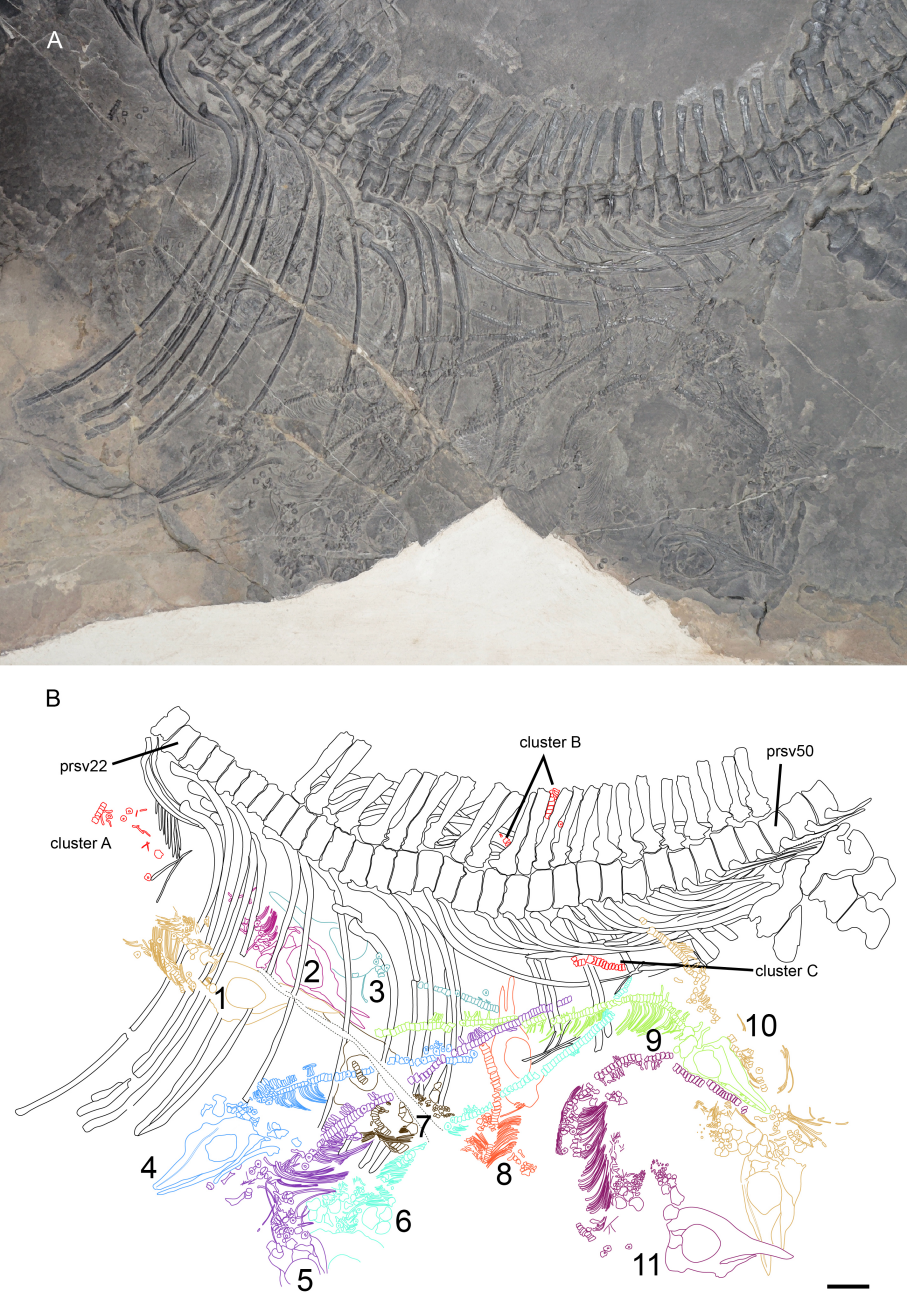
Fetuses and fetal clusters of *Mixosaurus panxianensis* ZMNH M8769. Photograph (A) and interpretive line drawing (B). Abbreviation: prsv, presacral vertebra. Scale bar is two cm.

Fetuses:

Eleven fetuses can be confidently identified within the specimen. They are exceptionally well-preserved and located between the ribs of the mid-posterior dorsal vertebrae (Figs. 1 and 5). The exquisite preservation within the abdominal cavity allows for the clear identification of these eleven fetal individuals, although the delicate and compressed nature of the remains makes the precise interpretation of each skeletal element within a single fetus challenging. Furthermore, several scattered and disarticulated vertebral series are present, potentially belonging to some of the fetuses or even more additional fetal individuals. The centra of fetuses are deeply amphicoelous in the manner characteristic of ichthyosaurs. Their notochordal foramina remain open, a definitive osteological indicator of immaturity consistent with reports in a fetal specimen of *Ichthyosaurus somersetensis* (Lomax & Sachs, 2017; Lomax et al., 2017). For descriptive purposes, these fetuses are numbered sequentially from fetuses 1 to 11 (Figs. 1 and 5), and detailed measurements and comparisons are provided in Table 1.

**Table 1:** Measurements (in mm) of the fetuses and fetal clusters in ZMNH M8769. Fetal skull orientation is relative to the adult skeleton.

Fetus no.	Overall length (mm)	Preserved skull length (mm)	Preserved vertebral column length (mm)	Number of vertebrae	Range of centrum diameters (mm)	Average of centrum diameters (mm)	Fetal skull orientation
1	103.5	56.5	29.2	26	1.5–2.9	2.3	posterior
2	83.3	53.6	12.9	6	2.4–2.9	2.7	posterior
3	112.9	57.7	41.4	26	2.0–3.2	2.7	anterior
4	155.9	51.1	98.0	51	1.7–3.6	2.7	anteroventral
5	191.1	20.5	139.5	24	2.0–4.3	3.1	anteroventral
6	186.3	16.2	155.8	67	1.3–3.5	2.7	anteroventral
7	98.4	45.4	61.7	28	2.3–3.6	2.8	ventral
8	95.7	56.9	53.5	33	1.6–4.2	3.1	dorsal
9	193.1	43.8	140.5	81	2.0–4.0	2.9	posterior
10	178.4	56.1	119.4	80	2.0–3.4	2.5	ventral
11	237.5	56.5	123.3	86	2.2–3.6	2.9	posteroventral
cluster A	/	/	/	10	2.2–2.9	2.5	/
cluster B	/	/	/	24	2.1–3.0	2.6	/
cluster C	23.9	/	/	16	2.3–3.6	2.9	/
cluster D	33.2	/	28.5	32	1.3–3.1	2.2	/

The orientation of a fetus is identified relative to that of the adult. Fetus 1 (Figs. 1 and 5) comprises a nearly complete skull oriented caudally, at least one forelimb, and 26 centra. Partially articulated ribs cover the series from the cervical to mid-dorsal region. Fetus 2 consists of a partial skull facing posteriorly and 6 centra. Fetus 3 preserves an incomplete skull oriented anteriorly and 26 centra. Fetus 4 is preserved in two sections: an anterior part containing a relatively complete skull and a continuous centra series, and a posterior part with a continuous series of centra, when the overall skeleton faces anteriorly. Fetus 5 includes an incomplete orbit with the post-orbit elements and a continuous vertebral column of at least 24 centra. Fetus 6 is represented by posterior skull fragments, discernible elements from both forelimbs, partial ribs, and at least 67 centra. Fetuses 4, 5, and 6 are preserved in near-parallel alignment. Situated among them is fetus 7, which preserves partial orbits, ribs, a humerus, some carpal elements, and approximately 28 vertebrae, along with other unidentifiable fragments. Fetus 8 preserves a relatively complete skull, partially discernible limb elements presumably from a forelimb, a continuous series of 33 centra, several rib fragments, and other skeletal debris. Remarkably, its cranium and postcranial skeleton are preserved in a 180-degree flexed orientation. This configuration suggests two kinds of alternative possibilities: either the skeleton underwent significant flexion during fossilization, or the cranial and postcranial components belong to two distinct individuals. Fetus 9 exhibits excellent preservation and is oriented posteriorly, including a nearly complete skull with discernible teeth embedded in the premaxilla, several rib fragments, and an extended and continuous series of 81 centra. Notably, some centra preserve neural arches and spines, indicating that these neural arches and tall spines had been developed in early stage before parturition in *Mixosaurus*. Fetus 10 preserved a relatively complete cranium, one forelimb, partial ribs, and a vertebral column bearing neural arches with tall spines too. Fetus 11 represents the most complete skeleton among the eleven fetal individuals, preserving a relatively complete cranium with at least four discernible teeth on the premaxilla, nearly intact both forelimbs, a fragmentary hindlimb, partial ribs, and a continuous vertebral column of at least 86 centra with associated neural arches with spines. Its body is curved, with the skull oriented posteriorly.

In addition to the eleven confirmed fetal specimens, four clusters A, B, C, and D (Figs. 1, 3 and 5) of disarticulated and displaced vertebral series are identified but remain unassigned. Cluster A comprises 10 centra and partial rib fragments, which are likely dorsal elements. Its location posterior to fetuses 1 and 2 suggests its most possible assignment to them. Cluster B comprises 24 scattered centra displaced dorsally to the neural spines of the adult skeleton, and their preservation characteristics may preclude an association with the aforementioned eleven fetuses. Cluster C contains 16 centra preserved dorsally to fetus 9. This series may represent either an additional fetal individual or displaced components from a known fetus, with fetus 10 being the most plausible source based on spatial proximity. Cluster D is displaced from the abdominal cavity and lies adjacent to the right scapula of the adult (Figs. 1 and 3). It represents continuous posterior caudal vertebrae with a possibly broken pelvic girdle, and it is most possibly assigned to fetus 1 or 2.

## Discussion

The general preservation of the specimen supports the interpretation that the eleven small ichthyosaurs represent fetuses rather than ingested prey. This conclusion is based on the following evidence: (1) most of the eleven fetal skeletons are predominantly located within the abdominal cavity of the adult individual; (2) the fetal vertebrae and ribs show no physical damage indicative of mastication or gastric processing; (3) there is no erosive trace from digestive secretions on the fetal remains; (4) no typical ichthyosaur stomach contents expected in a predation scenario, such as cephalopod hooks (Druckenmiller et al., 2014), are associated with the fetuses.

The fetal skeletons exhibit no pronounced disparity in size or development stage. The completely preserved skulls show highly comparable cranial lengths, while the vertebral centra diameters (both maximal and average) are consistent across individuals (Table 1). These morphometric uniformities suggest that the fetuses were simultaneously on an identical stage of ontogeny. All these fetal skeletons appear highly developed, indicating a late prenatal or perinatal state compared to a certain early ichthyosaur neonate (Lomax et al., 2017). Their dermatocranial elements seem well ossified, including an elongate snout of half the skull length being comparable to that in the adult, many small teeth on the jaws, the tight connections between nasal, frontal, and parietal within one half of the skull roof, when a slit-shaped non-classical fontanel can be observed in fetuses 4 and 10 in laterodorsal view (Fig. 5). Other parts on the skull, such as the chondrocranial bones of occiput, are likely less ossified compared to larger juveniles and adults, as the pattern documented in detail in *Mixosaurus cornalianus* (Miedema et al., 2023b) and *Stenopterygius quadriscissus* (Miedema & Maxwell, 2022). The vertebral columns are also well developed, with the centra closely abutted and no obvious notochordal openings. The ribs possess longitudinal ridges along their shafts and expanded proximal ends for the articulations to the vertebrae, similar to the adult condition (Fig. 5). In the fetuses 10 and 11, the preserved appendicular skeletons exhibit largely ossified girdle and limb elements, with minimal spacing between the bones. Based on cranial and vertebral development, the fetal individuals in ZMNH M8769 resemble those in the perinatal stage observed in *Stenopterygius quadriscissus* (Miedema & Maxwell, 2022; Miedema & Maxwell, 2026), and the fetuses in this gravid specimen suggest that *Mixosaurus* may be capable of birthing many offspring in a single reproductive event (Miedema et al., 2023a). The fetuses in this specimen exhibit inconsistent body orientations, and some of the fetuses (*e.g.*, the fetuses 8 and 11) appear to retain a curled posture (Fig. 5). According to the relative locations of the fetuses and the adult, fetuses 1 to 5 and even 6 seem to have remained almost fully within the uterus showing both head- (fetuses 1 and 2) and tail-first (fetuses 3 to 6) presentations, and fetuses 7 to 11 may have been scattered by gas explosion after death. Nevertheless, it suggests that neither a strict tail-first or head-first orientation was maintained during pregnancy or even at parturition, aligning with the conclusions of Miedema et al. (2023a).

**Figure 6:**
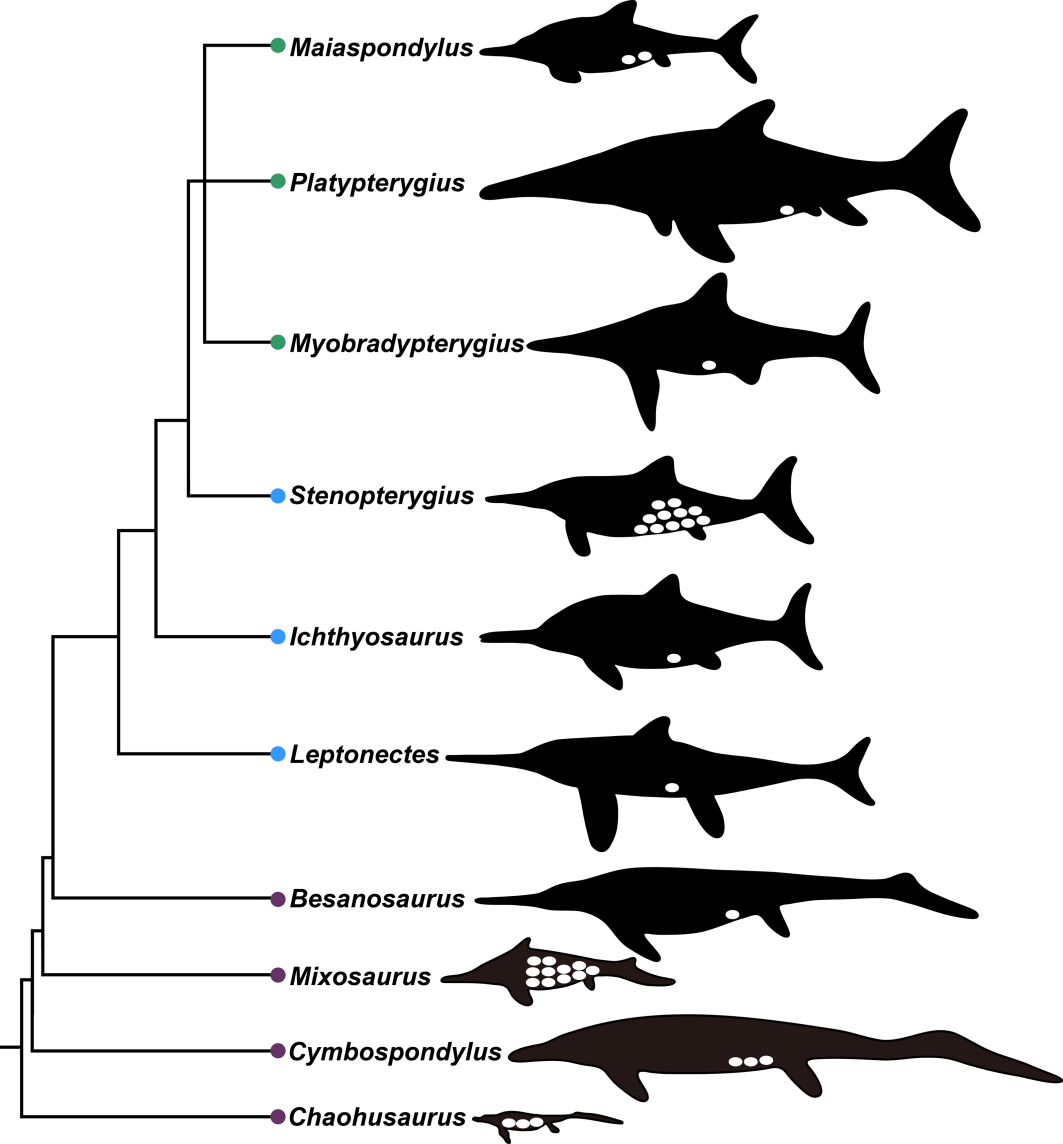
Cladogram of ichthyopterygians and the highest-recorded fetal counts. Taxa known with gravid specimens and fetal remains are listed, topology is modified from Bindellini et al. (2021). Relative body sizes of different taxa are showed but not strictly scaled, and the white dots represent fetal counts rather than fetal sizes. Triassic taxa with purple, Jurassic taxa with blue, and Cretaceous taxa with green tips in the cladogram.

Fossil evidence indicates that some post-Triassic ichthyosaurs bore several fetuses (Fig. 6), with the highest previously recorded number being eleven fetuses in an Early Jurassic *Stenopterygius* specimen Böttcher (1990). In contrast, earlier diverging Triassic ichthyosaurs, such as the gravid samples of *Chaohusaurus* from the Early Triassic China and *Mixosaurus* from the Middle Triassic Switzerland, were previously reported to carry no more than three fetuses (Brinkmann, 1996; Motani et al., 2014; Miedema et al., 2023a). The specimen described in this study, however, contains the highest number of fetuses ever recorded in a Triassic ichthyosaur and even all the ichthyosaur fossils hitherto known. The specimen of *Stenopterygius* with eleven fetuses (Böttcher, 1990) is probably an extreme case, when most of the gravid specimens of this genus exhibit the medium litter size with three or even one fetuses. Even though ZMNH M8769 could be an exceptional example, this discovery demonstrates that a high fetal carrying capacity during a single pregnancy, exceeding ten individuals, had evolved early in ichthyosaur evolution, as evidenced here in the Middle Triassic *Mixosaurus*. Furthermore, a high fecundity supports the plausible interpretation that *Mixosaurus* exhibits a reproductive strategy leaning toward r-selection rather than K-selection, characterized by high offspring numbers and presumably minimal parental investment, but more specimens with such a large number of fetuses are needed to further support this hypothesis. This r-selection strategy seems analogous to some small-sized eosauropterygians, such as *Keichousaurus hui* (Cheng, Wu & Ji, 2004), but contrasts with the K-selection strategy inferred for some other marine reptiles, such as some plesiosaurs, which produce fewer offspring accompanied by intensive parental care (O’Keefe & Chiappe, 2011). Notably, the number of preserved fetuses in *Stenopterygius* varies in different individuals, among which most gravid specimens remain one to three fetuses and a single specimen has eleven ones. It may suggest that the reproductive behavior of these ichthyosaurs could change due to other physical and environmental factors. *Mixosaurus* is among the most successful and widely distributed ichthyosaur genera of the Mesozoic, with abundant fossil remains recovered from both western and eastern Tethyan faunas (Fang, Wolniewicz & Liu, 2024; Jiang et al., 2006; Miedema et al., 2023a) as well as from broader global Triassic deposits (Engelschiøn et al., 2023; Schmitz et al., 2004). Compared with other ichthyosaurs, its high-productivity reproductive strategy may have contributed significantly to its evolutionary success and widespread prevalence in Triassic seas.

## Conclusions

Based on an exquisitely preserved specimen of *Mixosaurus panxianensis* from the Middle Triassic in southwestern China, we provide an anatomical description of both the adult and associated fetal skeletons. The description on the adult supplements the morphologic information for this genus and species, for which the original type material is incomplete (Jiang et al., 2006). The description of the fetuses further documents the morphology and size of late-stage prenatal individuals in this taxon. The principal findings and contributions of this work are threefold. First, we confirm the status of the fetuses based on their intra-uterine position, excellent articulation, absence of digestive erosion, and advanced degree of ossification, collectively indicating a late prenatal or perinatal developmental stage. Second, the inconsistent body orientations of the fetuses suggest that neither a strict head-first nor tail-first preference of birth presentation was maintained in mixosaurids even with such a large number of fetuses, and this observation aligns with recent hypotheses regarding parturition dynamics in other Triassic ichthyosaurs (Gingerich et al., 2009; Miedema et al., 2023a). Third, the large litter size suggests that *Mixosaurus* possibly employed the reproductive strategy more resembling r-selection, like in certain small-sized eosauropterygians (O’Keefe & Chiappe, 2011), characterized by high fecundity.

This study investigated the reproductive biology of early-diverging ichthyosaurs, focusing on litter size that was relatively less discussed in these Triassic ichthyosaurs in previous studies. This new specimen preserves at least eleven fetuses, representing the largest litter size recorded in any known ichthyosaur. It directly demonstrates that a high fetal carrying capacity (exceeding ten individuals) had evolved early in ichthyosaur history. Future discoveries of more gravid ichthyosaur fossils from the Triassic are needed to determine whether high fecundity was unique to *Mixosaurus* or a more widespread reproductive strategy among other early-diverging lineages of ichthyosaurs.

## Anatomical abbreviations

a: angular
ar: articular
ast: astragalus
atp: atlas pleurocentrum
axp: axial pleurocentrum
atn: atlas neural arch
axn: axial neural arch
cal: calcaneum
cap: caudal peak
cav: caudal vertebra
ch: chevron
cv: cervical vertebra
d: dentary
exn: external naris
f: frontal
fe: femur
fi: fibula
h: humerus
i: intermedium
il: ilium
is: ischium
I-V: first to fifth metatarsals
j: jugal
l: lacrimal
m: maxilla
meg: Meckelian cartilage grove
n: nasal
p: parietal
pb: pubis
pfm: parietal foramen
pi: pisiform
pm: premaxilla
po: postorbital
pof: postfrontal
prf: prefrontal
prsv: presacral vertebra
qj: quadratojugal
r: radius
ra: radiale
sa: surangular
sav: sacral vertebra
sc: scapula
sq: squamosal
st: supratemporal
ti: tibia
u: ulna
ul: ulnare
utf: upper temporal fenestra
1-4: first to fourth distal tarsals.

## Institutional abbreviations

GMPKU: Geological Museum of Peking University, Beijing, China
IVPP: Institute of Vertebrate Paleontology and Paleoanthropology, Beijing, China
SMNS: Staatliches Museum für Naturkunde Stuttgart, Stuttgart, Germany
ZMNH: Zhejiang Museum of Natural History, Hangzhou, China

## Additional Information and Declarations

### Data Availability

The following information was supplied regarding data availability: Specimen ZMNH M8769 is reposited at the Zhejiang Museum of Natural History, Hangzhou, China. Curator [Wei Wang] [wangwei2014@ivpp.ac.cn]
